# Sincere, Deceitful, and Ironic Communicative Acts and the Role of the Theory of Mind in Childhood

**DOI:** 10.3389/fpsyg.2017.00021

**Published:** 2017-01-30

**Authors:** Francesca M. Bosco, Ilaria Gabbatore

**Affiliations:** ^1^Department of Psychology, Center for Cognitive Science, University of TurinTurin, Italy; ^2^Neuroscience Institute of Turin, University of TurinTurin, Italy; ^3^Faculty of Humanities, Research Unit of Logopedics, Child Language Research Center, University of OuluOulu, Finland

**Keywords:** pragmatics, development, theory of mind, deceit, irony, direct and indirect speech acts

## Abstract

The aim of the study is to investigate the relationship among age, first- and second-order Theory of Mind and the increasing ability of children to understand and produce different kinds of communicative acts – sincere, ironic, and deceitful communicative acts – expressed through linguistic and extralinguistic expressive means. To communicate means to modify an interlocutor’s mental states (Grice, 1989), and pragmatics studies the inferential processes that are necessary to fill the gap, which often exists in human communication, between the literal meaning of a speaker’s utterance and what the speaker intends to communicate to the interlocutor. We administered brief video-clip stories showing different kinds of pragmatic phenomena – sincere, ironic, and deceitful communicative acts - and first- and second-order ToM tasks, to 120 children, ranging in age from 3 to 8 years. The results showed the existence of a trend of difficulty in children’s ability to deal with both linguistic and extralinguistic pragmatic tasks, from the simplest to the most difficult: sincere, deceitful, and ironic communicative acts. A hierarchical regression analysis indicated that age plays a significant role in explaining children’s performance on each pragmatic task. Furthermore, the hierarchical regression analysis revealed that first-order ToM has a causal role in explaining children’s performance in handling sincere and deceitful speech acts, but not irony. We did not detect any specific role for second-order ToM. Finally, ToM only partially explains the observed increasing trend of difficulty in children’s pragmatic performance: the variance in pragmatic performance explained by ToM increases between sincere and deceitful communicative acts, but not between deceit and irony. The role of inferential ability in explaining the improvement in children’s performance across the pragmatic tasks investigated is discussed.

## Introduction

Pragmatic ability refers to the use of language (Levinson, 1983) and other expressive means, such as non-verbal/extralinguistic means, i.e., gestures and body movements (Bara, 2010), to convey a specific meaning in a given context. Interesting examples of such ability are indirect speech acts, meaning acts through which the speaker communicates more than is literally said to the listener (Searle, 1975); deceitful communicative acts, meaning intentional attempts to manipulate the listener’s mental state in order to induce them to believe something untrue (Perner, 1991); and irony, meaning communicative acts expressing the opposite of what is meant by the speaker (Grice, 1989).

Evidence in the literature show that pragmatic ability correlates with Theory of Mind (ToM; Premack and Woodruff, 1978), i.e., the human ability to attribute mental states to oneself and to other individuals. Evidence also show that this ability increases during childhood (Wellman and Liu, 2004) and adolescence (Bosco et al., 2014, 2016; Brizio et al., 2015). Similarly, the increasing ability of children to manage these pragmatic phenomena as they grow older has been well documented in the literature. At around one year of age children start to understand and use direct speech acts, meaning utterances that express literally and exactly what the speaker intends to say (Searle, 1975), in order to communicate with another person (Garvey, 1984). However, children also become able to handle indirect speech acts early on in their development. Reeder (1980), for example, showed that starting from 2;6 years of age, children understand equally well, in an adequate context, that direct utterances like ‘I want you to do that’ and indirect requests like ‘Would you mind doing that?’ have the same conventional communicative meaning (see also Bernicot and Legros, 1987). Bucciarelli et al. (2003) also showed that, starting from 2;6 years of age, children understand direct and conventional forms of indirect speech acts (“Would you mind” “Do you know?”, etc) equally well, and that such ability increases with age (see Bosco and Bucciarelli, 2008).

Studies in the literature have shown that children’s ability to deal with verbal deceit also increases with age. In particular, starting from three years of age (Lewis, 1993; Bussey, 1999), children start using lies, meaning false utterances proffered with the intention of avoiding a disagreeable consequence such as a punishment (Leekman, 1992). Talwar and Crossman (2011) argue, in their review of the literature, that a child’s ability to lie could be considered normative, testifying the child’s social and cognitive development. The ability to handle lies of different complexity evolves during the pre-school to school period: as they grow up, children become able to consider the speaker’s intention and the impact of the social acceptability of lying and they start deceiving (for a review, see Talwar and Crossman, 2011). A deceitful communicative act is a speaker’s intentional attempt to manipulate the listener’s mental states in order to induce them to believe something untrue (Perner, 1991). Peskin (1996) claims that, in order to comprehend deceit, the speaker must take as shared with the listener something the speaker does not really believe. Peskin also claims that it is necessary to understand that the listener thus comes to hold a false belief, and observes how starting from the age of 4, children fully comprehend the speaker’s intention to deceive. The ability to deceive has thus been frequently explained on the basis of the ability to use a fully developed ToM (Chandler et al., 1989; Polak and Harris, 1999; Ma et al., 2015). Talwar et al. (2007, p. 804) for example affirm that “Lying, in essence, is ToM in action”; to deceive consists of creating a false-belief in the interlocutor’s mind (Lee, 2000). In particular, Talwar et al. (2007) investigated the relation between first- and second- order ToM and children’s ability to lie. First-order ToM involves the comprehension of another person’s belief about a certain state of the world, while second-order ToM involves the ability to infer what one person believes about another person’s thoughts, meaning to understand nested mental states (Perner and Wimmer, 1985). Talwar et al. (2007) reported a correlation between 6 and 11-year-old children’s second-order ToM and the ability to lie. Similarly, Cheung et al. (2015) found a correlation between 7 and 9-year-old children’s second-order ToM and their ability to understand a liar’s intention. The increasing ability of children to manage more complex forms of deceit has thus been explained on the basis of the children’s development from first-order to second-order ToM. However, the exact role of (first- and second-order) ToM in explaining a growing child’s ability to manage deceit is not yet completely clear (Talwar and Crossman, 2011). For example, some authors (Russell et al., 1995), claimed that not yet fully developed ToM abilities are not the best factor for explaining children’s difficulty in managing complex deceit, and proposed that the executive demand (in terms of executive functions as planning and shifting) that the comprehension of complex deceit requires is the best explanatory factor. Bosco and Bucciarelli (2008) also argued that ToM did not seem to be the best factor for explaining children’s ability to manage deceitful speech acts of increasing complexity.

Focusing now on irony, in its easiest form, this typically takes place when an utterance expresses the opposite of what the speaker means (Grice, 1989). In particular, irony involves a discrepancy between the literal meaning and the speaker’s communicative intent (Mey, 2001). Children usually start to develop the ability to recognize ironic speech acts between five and six years of age (Dews et al., 1996; Harris and Pexman, 2003; Filippova and Astington, 2010), although younger children may sometimes also understand irony (Loukusa and Leinonen, 2008; Angeleri and Airenti, 2014) and such ability improves over time (Demorest et al., 1984; Dews et al., 1996; Dews and Winner, 1997; Bosco and Bucciarelli, 2008; Filippova and Astington, 2008). Loukusa and Leinonen (2008), for example, found a significant difference between 6- and 7-year-olds in their ability to provide a correct explanation in a comprehension task on a simple ironic utterance, and Bosco and Bucciarelli (2008) reported that children of 6, 8, and 9 years of age found it easier to comprehend simple forms of irony, that is, utterances directly in contrast with the background knowledge, than complex ones, involving utterances implying knowledge that is in contrast with the background scenario.

According to Winner (1997), in order to interpret an ironic utterance correctly, the child must have the ability to detect incongruity or falsehood, to avoid mistaking irony for error, to understand another person’s beliefs, and to avoid interpreting irony as deception. In line with this theoretical proposal, Nilsen et al. (2011) showed that second-order ToM is correlated with children’s comprehension of verbal irony. Specifically, they pointed out that adults and older children aged between 8 and 10, but not younger children aged 6 to 7 years, were able to recognize that listeners require contextual knowledge to comprehend irony.

The studies in the literature have mainly focused on one single pragmatic phenomenon (and its possible relation with ToM) at a time, and only a few have undertaken a direct comparison of the different phenomena (Bosco and Bucciarelli, 2008; Bucciarelli et al., 2003). In particular, Bosco et al. (2012a, 2013) provided a broad assessment of the abilities of children ranging in age from 5 to 8;6 years, to comprehend and produce: direct and indirect speech acts (that the authors define as standard communication acts), and deceitful and ironic communication acts, using both linguistic and nonverbal/extralinguistic means of expression, such as gestures and body posture. The authors reported that the ability to perform all the pragmatic tasks investigated increases with age in children aged between 5 and 8;6 years, and their ability to deal with standard communication acts (direct and indirect), and deceitful and ironic speech acts also improves. In line with Bucciarelli et al. (2003), the authors explained the existence of such an increasing trend of difficulty on the basis of the Cognitive Pragmatic theory (Bara, 2010) and the increasing complexity of the inferential processes involved in the various pragmatic tasks investigated. The ability to infer refers to the cognitive capacity necessary to fill the gap, which often exists, between the literal meaning of an utterance and what the speaker actually means (Searle, 1975). According to the Cognitive Pragmatic theory, in expressing a sincere communicative act (direct and indirect communicative acts), the actor says something that is in line with his/her private beliefs. In terms of the inferential processes involved, the comprehension or production of a sincere communicative act merely requires the partner to refer to the background knowledge shared between the interlocutors. By contrast, the comprehension and production of deceitful and ironic communicative acts requires more complex inferential processes. In particular, in deceit, what the speaker says is in conflict with his private knowledge, but it does not contradict the knowledge given as shared with the partner. In a case of deceit, the partner has to recognize the difference between what is expressed and what the speaker privately entertains. In irony, the actor’s communicative intention is again in conflict with his private knowledge, as in the previous case, but it also contradicts the knowledge given as shared with the partner. This makes an ironic communicative act more difficult to entertain than a deceitful one (for a detailed description, see Bucciarelli et al., 2003; Bosco and Bucciarelli, 2008; Bara, 2010).

However, a possible different explanation for the increasing trend of difficulty in the comprehension and production of the pragmatic tasks described above implies a role for ToM, and in particular it states that ToM could play a greater role in deceitful communicative acts (Flanagan, 1992; Sodian and Frith, 1992) and ironic communicative acts (Happé, 1993) as compared to standard (direct and indirect) ones. Winner and Leekman (1991) assume that it is more difficult to understand irony than deceit because the former requires second-order ToM, whereas the latter only requires first-order ToM. In particular, Sullivan et al. (1995) found that starting from 7 years of age, children can distinguish lies from jokes, and they attribute this to the acquired ability to attribute second-order mental states.

To the best of our knowledge, no previous studies have empirically investigated such a hypothesis by assessing, in the same sample of participants, the possible role of first- and second-order ToM in explaining children’s increasing ability to comprehend and produce sincere (direct and indirect), deceitful, and ironic communicative acts. For this reason, the aim of the study was to investigate the increasing ability of children to manage different kinds of pragmatic phenomena, i.e., direct and indirect, deceitful, and ironic speech acts, and the possible role of first- and second-order ToM in explaining such performance. In detail, we wished to replicate the findings of Bosco et al. (2013, see also Bucciarelli et al., 2003; Bosco and Bucciarelli, 2008), and expected: (i) to find children’s performance on all the investigated tasks to improve with age; (ii) to find an increasing trend of difficulty in the comprehension and production of the investigated pragmatic phenomena, namely sincere communicative acts (direct and indirect), and deceitful and ironic communicative acts, in both the linguistic and non-verbal/extralinguistic modalities, including the use of gestures and body movements. In particular, the novelty of the present study was (iii) to explore the causal role of ToM (first- and second-order) in explaining such an improvement in their performance, in both the linguistic and non-verbal/extralinguistic modalities, within each investigated phenomenon. Moreover, (iv) we investigated the possible role of ToM in explaining the increasing trend of difficulty we expected to find across the various pragmatic phenomena investigated.

## Materials and Methods

### Participants

The sample consisted of 120 Italian children (60 males and 60 females) ranging in age from 3 to 8 years. In order to compare the subsamples’ performance in a more reliable way, the sample was organized in 4 age groups so that there was a one year difference between one age group and the next: Group A (3 years; 6 months – 4 years) (*M* = 3;10; *SD* = 0;2); Group B (5–5;6) (*M* = 5;3; DS = 0;2); Group C (6;6–7) (*M* = 6;10; DS = 0;2); Group D (8–8;6) (*M* = 8;3; DS = 0;3). Each age group was composed of 30 children and was balanced for gender, including an equal number of males and females.

The children were recruited from four different schools in the Piedmont area (Italy). Research assistants visited the schools before data collection commenced, and provided the teachers with details about the study. A letter containing all the details about the research was sent to the children’s families, together with an informed consent form, which the parents were required to complete. Only children whose parents gave their consent were included in the sample.

### Material

The experimental protocol consisted of a selection of 48 items taken from the linguistic and extralinguistic scales of the ABaCo (Sacco et al., 2008; Angeleri et al., 2012; Bosco et al., 2012a), a validated assessment tool to evaluate pragmatic abilities in typical (Bosco et al., 2013) and atypical (Angeleri et al., 2016) development. Examples of ABaCo items are provided in Appendix A.

For each expressive modality (i.e., linguistic and extralinguistic), the experimental task contained the same number and structure of items, and assessed the same type of pragmatic phenomena (half in comprehension and half in production):

- 8 sincere communicative acts, namely 4 direct and 4 indirect communicative acts;
- 8 deceitful communicative acts;
- 8 ironic communicative acts.

Each item consists of a video lasting 20–25 s, comprising a controlled number of words (range: 7 ± 2), and representing a communicative interaction between two people. The linguistic items investigate pragmatic phenomena expressed primarily through linguistic means, while the extralinguistic items are composed of communicative acts expressed through gestures (for a detailed description of the items, see Bosco et al., 2013).

In comprehension tasks, participants observed an interaction between two actors, and they were required to understand what was communicated (e.g., *In your opinion, what did the girl want to say to the boy?*). In production tasks, participants observed only the initial part of an interaction, and they were asked to produce a communicative act appropriate with respect to the proposed communicative situation (e.g., *The child doesn’t want to be discovered. What could he say?*).

For each pragmatic task, it was possible to obtain a score of “0” when the answer was considered incorrect and “1” when the answer was considered correct. More details concerning scoring criteria are reported in Appendix A (see also Sacco et al., 2008; Bosco et al., 2013). Inter-rater reliability was calculated using Cohen’s Kappa on the scores assigned to 40 randomly selected children (about 33% of the total sample): *K* was 0.67 (*p* < 0.001) 95% CI (0.653, 0.696), indicating substantial agreement (Landis and Koch, 1977).

In addition to the pragmatic tasks, a number of ToM tests were administered to the children. See Appendix B for a detailed description of the items.

#### Sally and Ann Task

In this task (Baron-Cohen et al., 1985), the child is required to observe a scene acted out by two paper dolls, Sally and Ann: *Sally places her ball in the basket and leaves the scene. Ann moves the ball from the basket to the box*. Then the child is required to reply to a test question (*When Sally comes back, where does she think the ball is?)* and a justification question (*Why does Sally think the ball is there?).* A score of 1 is gained when both the test and the justification questions are answered correctly.

#### Modified Smarties Task

This is a revised version of the original task developed by Perner et al. (1987). Because nowadays many children are no longer familiar with the famous candy brand, we introduced a packet of a currently famous brand of potato chips as the target object. During the task, the experimenter shows the packet of chips to every child and asks: *What is in there?* Then the experimenter opens the packet, showing that it contains pencils rather than the expected chips. The next question is: *What will someone else, who has not seen what the packet contains, think is in there, before it is opened?* A score of 1 is obtained when the child replies “chips” and a score of 0 is attributed to any other kind of answer.

#### John and Mary and Maxi Stories

These tasks (Sullivan et al., 1995) are a modified version of those used by Wimmer and Perner (1983) and Perner and Wimmer (1985) respectively, and they are told using cardboard puppets in order to reduce the memory load. The two stories assess second-order ToM, and they have an identical structure but different characters and settings. In the Maxi story, for example, the scenario is the following: *Maxi and Bobby are in their kitchen when their mother brings in some chocolate. Maxi would like to have some chocolate and his mom tells him he can have some after walking the dog. Unbeknownst to Maxi but not to Bobby, their mother takes the chocolate to the neighbor’s place. Unbeknownst to Bobby, Maxi discovers that their mom has taken the chocolate to the neighbor’s place. Bobby then goes to look for Maxi in the yard. His mother tells him that Maxi has gone to get some chocolate.* The task consists of a ‘second-order ignorance question’ (i.e., *Does Bobby know that Maxi knows where the chocolate is*?) and a ‘second-order belief question’ (i.e., *Where does Bobby go to look for Maxi*?). Along with the story-telling, a number of factual questions (e.g., *Why is Maxi in the yard?*) and first-order ToM questions (e.g., *Does Maxi know where the chocolate is now?*) were used to help the children to follow the storyline, but they were not taken into account in the scoring procedure. The children’s answers could be scored 1 (correct) or 0 (incorrect) for both second-order ignorance and belief questions. The mean value of the scores obtained from the two test questions was run to perform the analyses.

#### Picture Sequencing Task

Within the present study, just part of the original task (Langdon and Coltheart, 1999; Porter et al., 2008) was administered: the tasks used comprise six stories, including two social scripts (more than one person interacting in everyday social routines) and four false-belief sequences (a person, unaware of an event in a story, acts on a false belief). Internal consistency among these items was calculated (Cronbach’s alpha = 0.77). Each story was depicted in a set of four black-and-white picture cards. Two practice runs were used to allow the child to become familiar with the procedure of the task, and these were not considered in the scoring procedure. The set of cards for each story was placed face down in front of the child, and the child was required to arrange the cards in the correct order to tell the story according to the logical sequence of events, like in a comic-strip. Scores ranged from 0–6; each sequence scored 2 points if the first card was in the correct position, 2 points if the last card was in the correct position, and 1 point for each of the second and third cards being in the correct positions. Failure to produce a sequence was scored as 0.

Inter-rater reliability was calculated using Cohen’s Kappa also for ToM tasks scores attributed by two raters in about 33% of the total sample: Sally and Ann task, Modified Smarties task, John and Mary and Maxi Stories task. It was not calculated on the Picture Sequencing task scores, because the scoring procedures for this test only involve comparing the order of the sequences provided by the child with the correct ones provided by the test instructions. Since no different interpretation is possible, we did not consider it necessary to have a second rater for such scores. *K* ranged from 0.76 to 1 (*p* < 0.001), indicating substantial to almost perfect agreement (Landis and Koch, 1977).

### Procedure

The experimenters visited the schools before the beginning of the study, in order to familiarize with the children. The children dealt with the experimental tasks in a single individual session, lasting approximately 50 min and performed in a quiet room at the school. The video-taped stories were shown to the children one at a time, using a portable computer, and each session was video-recorded, to allow oﬄine coding procedures. The tasks were presented in two different random orders, A and B; the participants in each group were balanced for age and gender, and were assigned to order A or B of the protocol in a balanced way. The ToM tests were also balanced, so that they were presented to half of the participants before the presentation of the pragmatic protocol and to half of the participants after the presentation of the pragmatic protocol. Moreover, the ToM tasks were presented in two different random orders (first-order tasks followed by second-order tasks and vice versa). When performing the analyses, first- and second-order ToM scores were considered separately. In particular, the first-order ToM value was obtained using the average scores gained from the Sally and Ann, Smarties, and Picture Sequencing tasks. Likewise, the second-order ToM value was obtained by combining the average scores obtained from the John and Mary and Maxi tasks.

### Data Analysis

The distribution of scores for each kind of task was not normal in most age groups. In particular, the Kolmogorov–Smirnov test showed the distribution to be normal only in a few cases, namely extralinguistic deceit in group B and extralinguistic irony in both groups C and D, while data were not normally distributed in any of the other cases: linguistic sincere acts 0.001 *< p <* 0.011; linguistic deceit 0.001 < *p* < 0.042; linguistic irony: 0.001 < *p* < 0.027; extralinguistic sincere acts *p* < 0.001; extralinguistic deceit: 0.001 < *p* < 0.200; extralinguistic irony 0.001 < *p* < 0.181. We also performed a Shapiro–Wilk test, which confirmed the previous results: the distribution of scores was normal in only a few cases, namely linguistic irony in group D, extralinguistic deceit in group B, and extralinguistic irony in groups C and D; data were, instead, not normally distributed in any of the other cases: linguistic sincere acts.001 < *p* < 0.022; linguistic deceit 0.001 < *p* < 0.010; linguistic irony 0.001 < *p* < 0.164; extralinguistic sincere acts 0.002 < *p* < 0.010; extralinguistic deceit 0.001 < *p* < 0.178; extralinguistic irony 0.001 < *p* < 0.214. We thus conducted an arcsine transformation on the children’s answers in each pragmatic task (linguistic and extralinguistic sincere communicative acts, deceit, and irony) and each ToM task (first- and second-order). We were thus able to perform parametric analyses while satisfying the required assumptions.

To investigate children’s performance in managing different kinds of pragmatic tasks, we conducted a multivariate analysis of variance (MANOVA), with age as between-subject factor (type of age group: Group A 3;6–4, Group B 5–5;6, Group C 6;6–7, and Group D 8–8;6) and performance at sincere communicative act, deceit, and irony as dependent variables on both the linguistic and the extralinguistic scales. Analogously, children’s ability to manage different kinds of ToM tasks was investigated by conducting a MANOVA with age as between-subject factor (type of age group: Group A 3;6–4, Group B 5–5;6, Group C 6;6–7, and Group D 8–8;6) and performance at first- and second-order ToM tasks as dependent variables.

Moreover, in order to investigate the effect of performance on the different pragmatic tasks within each age group (type of pragmatic phenomena: sincere, deceit, irony), we performed separate ANOVA analyses, for both linguistic and extralinguistic tasks.

In order to investigate the correlation between pragmatic and ToM ability, we calculated the partial correlation (Pearson’s *r*, controlling for age) between children’s performance on pragmatic and ToM tasks in the overall sample.

Lastly, in order to investigate the specific effect of age and of first- and second-order ToM in explaining children’s pragmatic performance, we conducted a hierarchical regression analysis, including three steps: Age (step1), first-order ToM (step2) and second-order ToM (step3). Such variables were entered into the regression model as predictors to detect their impact on children’s performance on the pragmatic tasks (i.e., sincere, deceit and irony). Statistically significant correlations were found between linguistic and extralinguistic performance on the different types of tasks: sincere (*r* = 0.29; *p* = 0.001), deceit (*r* = 0.74; *p* < 0.001), irony (*r* = 0.63; *p* < 0.001). For this reason, and since the trends in scores were the same for both modalities in all age groups, in this regression analysis we collapsed the scores obtained for the linguistic and extralinguistic tasks into a single *type of pragmatic task score.* Despite the differences implied in these pragmatic phenomena, collapsing them into a single score provides a more statistically robust measure of overall pragmatic ability (Cronbach’s alpha = 0.93).

## Results

The scores obtained by each age group on the pragmatic and ToM tasks are summarized in **Tables 1** and **2**.

**Table 1:** Performance of each age group at the pragmatic tasks, mean (standard deviation).

	Linguistic Scale			Extralinguistic Scale		
	Sincere	Deceit	Irony	Sincere	Deceit	Irony
**Age Group**						
A (3;6–4)	0.75 (0.18)	0.34 (0.30)	0.15 (0.17)	0.44 (0.28)	0.19 (0.19)	0.06 (0.11)
B (5–5;6)	0.73 (0.16)	0.70 (0.30)	0.38 (0.23)	0.64 (0.26)	0.53 (0.30)	0.28 (0.30)
C (6;6–7)	0.79 (0.13)	0.93 (0.10)	0.45 (0.24)	0.84 (0.12)	0.81 (0.17)	0.43 (0.28)
D (8–8;6)	0.83 (0.12)	0.91 (0.12)	0.49 (0.22)	0.83 (0.13)	0.88 (0.13)	0.47 (0.28)
Overall sample	0.77 (0.15)	0.72 (0.33)	0.37 (0.25)	0.68 (0.27)	0.60 (0.34)	0.31 (0.30)

**Table 2:** Performance of each age group at the Theory of Mind (ToM) tasks.

	First-order ToM	Second-order ToM	ToM overall
**Age Group**			
A (3;6–4)	0.29 (0.25)	0.30 (0.29)	0.29 (0.23)
B (5–5;6)	0.53 (0.29)	0.40 (0.25)	0.46 (0.22)
C (6;6–7)	0.74 (0.24)	0.54 (0.25)	0.64 (0.20)
D (8–8;6)	0.80 (0.17)	0.62 (0.28)	0.71 (0.17)
Overall sample	0.59 (0.31)	0.47 (0.29)	0.53 (0.26)

In **Tables 3** and **4** the correlation coefficients among pragmatic tasks and ToM tasks, respectively, are provided.

**Table 3:** Correlations of pragmatic tasks scores.

Variables	1	2	3	4	5	6
(1) Linguistic scale - Sincere	–					
(2) Linguistic scale – Deceit	0.28^∗∗^	–				
(3) Linguistic scale – Irony	-0.01	0.51^∗∗^	–			
(4) Extralinguistic scale – Sincere	0.38^∗∗^	0.77^∗∗^	0.35^∗∗^	–		
(5) Extralinguistic scale – Deceit	0.21^∗^	0.81^∗∗^	0.59^∗∗^	0.69^∗∗^	–	
(6) Extralinguistic scale – Irony	-0.04	0.45^∗∗^	0.65^∗∗^	0.37^∗∗^	0.57^∗∗^	–

^∗∗^*p < 0.01; ^∗^*p* < 0.05*.

**Table 4:** Correlations of I and II order ToM tasks.

Variables	1	2	3	4	5
(1) Sally & Ann task; First-order ToM	–				
(2) Smarties task; First-order ToM	0.39^∗∗^	–			
(3) Picture Sequencing task; First-order ToM	0.43^∗∗^	0.32^∗∗^	–		
(4) Ice Cream Story task. Second-order ToM	0.44^∗∗^	0.23^∗^	0.30^∗∗^	–	
(5) Maxy task. Second-order ToM	0.22^∗^	0.38^∗∗^	0.39^∗∗^	0.39^∗∗^	–

^∗∗^*p < 0.01; ^∗^*p* < 0.05*.

On the linguistic scale, the MANOVA revealed a significant effect of age on the pragmatic performance [*F*_(9,348)_ = 8.97; *p* < 0.001; η^2^ = 0.19]. Separate univariate ANOVAs on the outcome variables revealed a significant effect of age on deceits [*F*_(3,116)_ = 36.77; *p* < 0.001; η^2^ = 0.49] and ironies [*F*_(3,116)_ = 12.09; *p* < 0.001; η^2^ = 0.24] but a not significant effect on sincere communicative acts [*F*_(3,116)_ = 2.12; *p* = 0.10; η^2^ = 0.05]. *Post hoc* pairwise comparison (Bonferroni) between the performance of A vs. B, B vs. C and C vs. D age group at each pragmatic task highlighted the following results: no differences were detected among the groups at the sincere acts (*p* = 1.0); the groups performed significantly differently at the deceitful acts (*p* < 0.001), with the only exception being Group C vs. Group D, which showed no differences (*p* = 1.0); finally, at the ironic tasks, a significant difference was found between Group A and B (*p* = 0.003), while no differences were detected between the remaining groups (0.999 < *p* < 1.0).

In terms of the extralinguistic scale, the MANOVA revealed a significant effect of age on the pragmatic performance [*F*_(9,348)_ = 10.11; *p* < 0.001; η^2^ = 0.21]. Separate univariate ANOVAs on the outcome variables revealed a significant effect of age on all the communicative acts investigated: sincere [*F*_(3,116)_ = 20.51; *p* < 0.001; η^2^ = 0.35], deceits [*F*_(3,116)_ = 48.63; *p* < 0.001; η^2^ = 0.56] and ironies [*F*_(3,116)_ = 13.18; *p* < 0.001; η^2^ = 0.25]. *Post hoc* pairwise comparison (Bonferroni) between the performance of each age group at each pragmatic task highlighted the following results: the groups performed significantly differently at the sincere (0.003 < *p* < 0.016) and deceitful acts (*p* < 0.001), with the only exception being Group C vs. Group D, which showed no differences both at sincere (*p* = 1.0) and at deceitful acts (*p* = 0.863); for what concerns ironies, again a significant difference was found between Group A and B (*p* = 0.033), while no differences were detected between the remaining groups (0.235 < *p* < 1.0).

The ANOVA analyses performed within each age group separately and concerning the linguistic tasks, revealed an effect of the type of task in all age groups [20.09 < *F*_(2,58)_ < 57.06; *p* < 0.001; 0.41 < η^2^ < 0.66]. Moreover, introducing contrasts for each analysis, we detected a linear contrast, depending on the type of pragmatic task in each age group [25.39 < *F*_(1,29)_ < 145.53; *p* < 0.001;.47 < η^2^ < 0.83]. The same pattern of results was found concerning extralinguistic tasks: an effect of the type of task was detected in all age groups [14.21 < *F*_(2,58)_ < 36.48; *p* < 0.001; 0.33 < η^2^ < 0.56] and contrast analysis revealed a linear contrast, depending on the type of pragmatic task in each age group [24.60 < *F*_(1,29)_ < 39.10; *p* < 0.001; 0.46 < η^2^ < 0.61].

In terms of children’s ability to manage different kinds of ToM tasks, the MANOVA revealed a significant effect of age on the children’s performance [*F*_(6,232)_ = 10.86; *p* < 0.001; η^2^ = 0.22]. Separate univariate ANOVAs on the outcome variables revealed a significant effect of age on first-order ToM performance [*F*_(3,116)_ = 27.60; *p* < 0.001; η^2^ = 0.42] as well as on second-order ToM performance [*F*_(3,116)_ = 7.37; *p* < 0.001; η^2^ = 0.16]. *Post hoc* pairwise comparison (Bonferroni) between the performance of each age group at first-and second order ToM tasks revealed the following results: the groups performed significantly differently at the first-order ToM tasks (0.003 < *p* < 0.005) with the only exception being Group C vs. Group D, which showed no differences (*p* = 1.0); at the second-order tasks, no differences were found between the performance of the age groups (0.512 < *p* < 1.0).

Partial correlation coefficients between linguistic and extralinguistic pragmatic tasks (comprehension and production ability) and overall ToM ability (first- and second-order tasks) are reported in **Table 5**.

**Table 5:** Partial correlation (Pearson *r*, controlling for age) between overall ToM tasks (first- and second-order) and pragmatic tasks, in the overall group.

	Linguistic Scale			Extralinguistic Scale		
	Sincere	Deceit	Irony	Sincere	Deceit	Irony
First-order ToM tasks	0.14	0.30^∗∗^	0.18^∗^	0.19^∗^	0.20^∗^	–0.02
Second-order ToM tasks	0.12	0.19^∗^	0.14	0.16	0.14	–0.07
Overall ToM	0.15	0.32^∗∗^	0.20^∗^	0.25^∗^	0.23^∗^	–0.05

^∗∗^*p < 0.01; ^∗^*p* < 0.05*.

As shown in **Table 5**, we found a significant correlation between overall ToM tasks and all the pragmatic tasks investigated, in both the Linguistic and Extralinguistic scales, with the only exception of sincere linguistic communicative acts and Extralinguistic irony. The same result applies for first-order ToM tasks. By contrast, the only significant relation we detected for second-order ToM tasks was between linguistic deceit and second-order ToM.

**Table 6** displays the results of multiple hierarchical regression analysis on the overall sample. In particular it shows all the coefficients of the regression models as well as the information about the summary of the model: adjusted regression coefficients (*R*^2^_Adj._) for each predictor variable, the change in *R*^2^ after the addition of first- and second-order ToM (*R*^2^_Change_), the change in *F* (*F*_Change_), and its significance value (Sig. *F*_Change_).

**Table 6:** Hierarchical regression analysis: pragmatic tasks (linguistic and extralinguistic) in the overall group.

DVs	IVs	*B*	*SE B*	β	*t*	*p*	*R^2^*	*R*^2^_Adj_	*R*^2^_Change_	*F*_Change_	Sig. *F*_Change_
**Pragmatic task**											
Sincere	Step 1										
	-Age	0.007	0.001	0.517	6.554	0.001	0.267	0.261	–	42.952	**0.001**
	
	Step 2										
	-Age	0.005	0.001	0.366	3.743	0.001	0.304	0.292	0.037	6.291	**0.014**
	-First-order ToM	0.157	0.063	0.245	2.508	0.014					
	
	Step 3										
	-Age	0.005	0.001	0.351	3.549	0.001	0.310	0.292	0.006	0.962	0.329
	-First-order ToM	0.138	0.066	0.215	2.100	0.038					
	-Second-order ToM	0.057	0.058	0.086	0.981	0.329					

Deceit	Step 1										
	-Age	0.017	0.002	0.725	0.725	0.001	0.526	0.522	–	130.858	**0.001**
	
	Step 2										
	-Age	0.014	0.002	0.586	0.586	0.001	0.558	0.550	0.032	8.457	**0.004**
	-First-order ToM	0.255	0.088	0.227	0.227	0.004					
	
	Step 3										
	-Age	0.014	0.002	0.570	0.570	0.001	0.565	0.553	0.007	1.837	0.178
	-First-order ToM	0.218	0.092	0.194	0.194	0.019					
	-Second-order ToM	0.110	0.081	0.094	0.094	0.178					

Irony	Step 1										
	-Age	0.008	0.001	0.514	6.508	0.001	0.264	0.258	–	42.349	**0.001**
	
	Step 2						*0.269*	0.257	0.005	0.829	0.364
	-Age	0.007	0.001	0.458	4.570	0.001					
	-First-order ToM	0.064	0.070	0.091	0.910	0.364					
	
	Step 3										
	-Age	0.007	0.002	0.457	4.486	0.001	0.269	0.250	0.000	0.006	0.938
	-First-order ToM	0.062	0.074	0.089	0.842	0.402					
	-Second-order ToM	0.005	0.065	0.007	0.077	0.938					

*Variables significantly predicting pragmatic performance is marked in bold*.

The regression analysis revealed that age (Step 1) explains 26% of the variance in children’s performance on sincere tasks, 53% on deceitful tasks and 26% on ironic tasks. The model also including children’s performance on first-order ToM tasks as a regressor (Step 2) only significantly improved the prediction for sincere (i.e., direct and indirect communicative acts) and deceitful tasks, but not for ironic ones. The addition of scores for performance on second-order ToM tasks (Step 3) did not improve the prediction for any of the pragmatic tasks.

As a final point, the analyses also showed that within both the model including first-order ToM (Step 2) and the model comprising second-order ToM (Step 3), *R*^2^ only partially follows the trend of increasing difficulty exhibited by children in solving pragmatic problems, when considering both linguistic and extralinguistic tasks, i.e., first-order ToM (sincere, *R*^2^ = 0.304; deceit, *R*^2^ = 0.558; irony *R*^2^ = 0.269), second-order ToM (sincere, *R*^2^ = 0.310; deceit, *R*^2^ = 0.565; irony *R*^2^ = 0.269). In particular, the *R*^2^ value increases across tasks between sincere and deceitful communicative acts, but not between deceitful and ironic ones.

## Discussion

The goal of the present study was to investigate the possible role of ToM – both first- and second-order - in explaining children’s ability to comprehend and produce different kinds of pragmatic phenomena, namely sincere (direct and indirect), deceitful, and ironic communicative acts, expressed through linguistic and non-verbal/extralinguistic modalities.

First of all, and in line with our expectation and the relevant literature (Bucciarelli et al., 2003; Bosco and Bucciarelli, 2008; Filippova and Astington, 2010; Talwar and Crossman, 2011; Bosco et al., 2013), overall our results showed that children’s ability to comprehend and produce the pragmatic phenomena investigated increases with age, in both the linguistic and non-verbal/extralinguistic modalities. Analyzing deeper this result for each pragmatic task and comparing age groups we found that, for the linguistic modality, children showed no differences at the sincere acts, while performed significantly differently at the deceitful acts with the only exception of oldest groups of age of 6- vs. 8- year-olds children; for what concern ironic acts, younger group of 3- year-olds children showed a significant worse performance than all the other groups, while children belonging to the remaining age groups had comparable performance. We explain such results on the base of the Cognitive Pragmatic theory, proposing that, because of the inferential process involved, sincere communicative acts are the easiest task to solve for children and thus they performed quite well starting from 3;6–4 years of age. Always following the tenets of the Cognitive Pragmatic theory a deceitful communicative act represents a more difficult pragmatic task to solve and only starting from 6;6–7 years of age children handle it without errors. Finally, irony is the most difficult task to solve and it represents a really hard task to manage for children as young as 3;6–4 years of age. However, it remains a quite difficult task also for the older children. Globally considered the same pattern of results and the same explanation hold for the extralinguistic modality; the only exception is represented by the younger 3- and 5- years-olds, who showed differences in performance at sincere communicative acts. A possible explanation for this difference is that the extralinguistic modality was harder for 3- and 5- year-olds children to deal with and this additional difficulty allowed this difference in performance to emerge.

In line with our hypothesis, and considering each age group separately, we also found an increasing trend of difficulty in children’s performance across the pragmatic tasks investigated: children were able to comprehend and produce sincere communicative acts more accurately than deceit, which was followed by ironic speech acts, which were the most difficult task to deal with. Considered overall, this linear increase in difficulty holds in both the linguistic and extralinguistic modality following the patterns of results found in previous studies (Bucciarelli et al., 2003; Bosco and Bucciarelli, 2008; Bosco et al., 2013).

The novelty of the present research was to explore the causal role of age and ToM – both first- and second-order - in explaining children’s pragmatic performance, in both the linguistic and non-verbal/extralinguistic modalities. Some authors have indeed proposed that pragmatics/communicative ability involves mentalizing, i.e., ToM, abilities (Sperber and Wilson, 2002; Tirassa et al., 2006a,b; Tirassa and Bosco, 2008; Fernandez, 2011; Bosco et al., 2012b; Cummings, 2015). In line with this proposal we found a correlation, controlling for age, between overall ToM tasks (first- and second-order tasks) and linguistic and extralinguistic irony and deceit, but not between linguistic sincere communicative acts and extralinguistic irony. The same pattern of results holds for first-order ToM. Considering second-order ToM, we only found a significant correlation with linguistic deceit.

The correlation we found between children’s performance on ToM tasks (overall and first-order) and sincere acts may be considered a surprising result. We can explain this result by considering that half of the items making up our experimental material were indirect communicative acts. Studies in the literature have suggested that ToM has a role in the comprehension of indirect speech acts. For example, Corcoran et al. (1995) and Corcoran (2003) showed that patients with schizophrenia, a disorder explained (e.g., Frith, 1994) on the basis of a primary deficit in ToM, have difficulties in the comprehension of indirect speech acts.

Our results concerning the correlation between ToM (overall and first-order) and deceit are in line with the current literature (see for example Chandler et al., 1989; Polak and Harris, 1999; Ma et al., 2015). In particular, our result regarding the significant role played by second-order ToM in dealing with deceitful acts is in line with Talwar and Lee (2008). The authors showed that the performance of children aged from 3 to 8 years on second-order ToM tasks is related to their ability to maintain a plausible explanation in order to not reveal their lies. Some authors also found that second-order ToM ability correlates with pro-social lies (Cheung et al., 2015; Williams et al., 2016), which are considered more sophisticated than lies. In particular, Broomfield et al. (2002) found that only pro-social, but not other forms of lies, are related to second-order ToM. However, our experimental material did not include pro-social lies, so a direct comparison is not possible. Our results concerning the correlation between ToM (second-order) and irony are also consistent with the literature, in particular with Winner (1997), who argued for the role of second-order ToM in irony comprehension and Nilsen et al. (2011), who reported that second order ToM is correlated with children’s comprehension of verbal irony.

Taken globally, our results are also in line with the literature concerning autism, a pathology characterized by a ToM impairment (Baron-Cohen et al., 1985) and showing how people with autism have difficulties in comprehending and producing indirect, deceitful and ironic communicative acts (Happé, 1993; Angeleri et al., 2016, for a review see Loukusa and Moilanen, 2009).

However, in order to conduct an in-depth investigation of the possible role of age and of first- and second-order ToM in explaining the improvement in children’s performance across each pragmatic task (linguistic and non-verbal/extralinguistic), we performed a hierarchical multiple regression analysis. We found that, as expected, age has a significant role in explaining children’s performance on all the investigated tasks. The results also showed, consistently with the correlation analysis, a significant role for first-order ToM in explaining children’s performance in the comprehension and production of sincere (direct and indirect) communicative acts as well as their ability to manage deceitful communicative acts. By contrast, we did not detect any significant role for second-order ToM in explaining any of the pragmatic tasks investigated, thus testifying, when the role of age and first-order ToM is kept under control, a limited causal role of this more sophisticated ToM aspect in explaining children’s ability to deal with sincere, deceitful and ironic communicative acts.

A direct comparison of this result with the current literature is not possible, since other studies (see for example Talwar and Lee, 2008; Nilsen et al., 2011) usually limit their investigation to the correlation analyses. An exception is the study by Angeleri and Airenti (2014) where, despite the significant correlation found between ToM (first and second-order) ability and the comprehension of linguistic ironic tasks, a more detailed investigation, run through path analysis, underlined that ToM had no direct effect on humor comprehension. In line with the results provided by Angeleri and Airenti (2014), our hierarchical regression analysis showed that, when the role of age is kept under control, neither first- nor second-order ToM has a direct impact on children’s performance on irony tasks. Our finding thus did not provide empirical support to theories proposing that ToM (Happé, 1993) and specifically second-order ToM (Winner and Leekman, 1991), plays a key role in explaining irony comprehension. Furthermore, the results of the present investigation, in addition to those of Angeleri and Airenti (2014) indicate that to use ironic statements - it is for example the case of some items composing the Strange Stories (Happé, 1994) – could not be not a reliable measure to investigate ToM ability in children.

Lastly, we now wish to focus on the role of ToM in explaining the increasing trend of difficulty shown by children in dealing with sincere, deceitful and ironic communicative acts, using both Linguistic and Extralinguistic expressive means. We found that ToM, neither first- or second- order, could be considered the best factor explaining our incresasing trend of difficulty in children’s performance. Indeed, we found that *R*^2^ only partially follows the trend of increasing difficulty exhibited by children in solving each kind of investigated task, i.e., sincere, deceitful and ironic communicative acts. The *R*^2^ value indicates how much variance is explained by a certain variable. If ToM (both first- and second-order) was the factor that best explained the difference in difficulty among the three tasks, we would expect the *R*^2^ value to increase according to the level of difficulty detected in managing linguistic and extralinguistic sincere communicative acts, deceit, and irony. However, this value increases when considering sincere and deceitful communicative acts, but not when considering deceit and irony.

To summarize, our results on the existence of an increasing trend of difficulty across pragmatic tasks seem only partially explained by the role of ToM (see also Bosco and Gabbatore, 2017). Considered overall, our results suggest a role for first-order ToM in explaining the differences in performance only when considering sincere and deceitful acts, but not when considering deceit and irony. A possible alternative explanation for the existence of such an increasing trend of difficulty is based on the inferential complexity underlying the pragmatic tasks investigated (see Bucciarelli et al., 2003; Bosco and Bucciarelli, 2008; Bosco et al., 2013). The existence of an increasing trend of difficulty in the comprehension and production of sincere (direct and indirect), deceitful, and ironic communicative acts has been experimentally demonstrated, not only in studies on children (see Bosco et al., 2009, 2012c), but also through the assessment of pragmatic abilities in patients with schizophrenia (Colle et al., 2013), and individuals with brain injury (Bara et al., 2001; Angeleri et al., 2008), left brain damage (Gabbatore et al., 2014), and right brain damage (Parola et al., 2016). Other authors in the literature have also highlighted the key role that the inferential processes play in the comprehension process (Leinonen et al., 2000). In particular Pexman and Glenwright (2007) highlighted the role of inferential ability in the comprehension of an ironic statement.

A limit of the present investigation is that it does not consider the role that other cognitive functions, such as executive functions like planning, working memory, inhibition, and shifting, might play in explaining the development of children’s communicative-pragmatic performance. In future, it might be useful to conduct a longitudinal study in order to observe the development of pragmatic abilities in a specific group of children over time. Even though the present investigation focuses on pragmatics, a further interesting topic of study is the influence of linguistic development on children’s ToM ability. As a final point, the merit of the present study was to help to clarify the (limited) causal role of first- and second-order ToM in explaining the improvement in children’s pragmatic performance across different kinds of pragmatic tasks, such as sincere, deceitful, and ironic communicative acts.

## Ethics Statement

Bio-ethical Committee of the University of Turin (Protocol no. 13620121).

## Author Contributions

IG took care of the preparation and administration of the experimental material, run the statistical analysis and wrote the corresponding part of the paper (Methods and Results). FB is responsible for the whole research project. She took care of the review of the literature and wrote the introductive part of the manuscript and its discussion.

## Conflict of Interest Statement

The authors declare that the research was conducted in the absence of any commercial or financial relationships that could be construed as a potential conflict of interest.

## References

[B1] Angeleri R. Airenti G. (2014). The development of joke and irony understanding: A study with 3-to 6-year-old children. *Can. J. Exp. Psychol.* 68 133–146. 10.1037/cep0000011 24364812

[B2] Angeleri R. Bosco F. M. Gabbatore I. Bara B. G. Sacco K. (2012). Assessment battery for communication (ABaCo): normative data. *Behav. Res. Methods* 44 845–861. 10.3758/s13428-011-0174-9 22180102

[B3] Angeleri R. Bosco F. M. Zettin M. Sacco K. Colle L. Bara B. G. (2008). Communicative impairment in traumatic brain injury: a complete pragmatic assessment. *Brain Lang.* 107 229–245. 10.1016/j.bandl.2008.01.002 18267340

[B4] Angeleri R. Gabbatore I. Bosco F. M. Sacco K. Colle L. (2016). Pragmatic abilities in children and adolescents with autism spectrum disorder: a study with the ABaCo battery. *Minerva Psichiatr.* 57 93–103.

[B5] Bara B. G. (2010). *Cognitive Pragmatics: The Mental Processes of Communication*. Cambridge: MIT Press.

[B6] Bara B. G. Cutica I. Tirassa M. (2001). Neuropragmatics: extralinguistic communication after closed head injury. *Brain Lang.* 77 72–94. 10.1006/brln.2000.2430 11247657

[B7] Baron-Cohen S. Leslie A. Frith U. (1985). Does the autistic child have a theory of mind? *Cognition* 21 37–46. 10.1016/0010-0277(85)90022-8 2934210

[B8] Bernicot J. Legros S. (1987). Direct and indirect directives: what do young children understand? *J. Exp. Child Psychol.* 43 346–358. 10.1016/0022-0965(87)90012-9

[B9] Bosco F. M. Angeleri R. Colle L. Sacco K. Bara B. G. (2013). Communicative abilities in children: an assessment through different phenomena and expressive mean. *J. Child Lang.* 40 741–778. 10.1017/S0305000913000081 23651672

[B10] Bosco F. M. Angeleri R. Zuffranieri M. Bara B. G. Sacco K. (2012a). Assessment battery for communication: development of two equivalent forms. *J. Commun. Disord.* 45 290–303. 10.1016/j.jcomdis.2012.03.002 22483360

[B11] Bosco F. M. Bono A. Bara B. G. (2012b). Recognition and repair of communicative failures: the interaction between theory of mind and cognitive complexity in schizophrenic patients. *J. Commun. Disord.* 145 181–197. 10.1016/j.jcomdis.2012.01.005 22402250

[B12] Bosco F. M. Bucciarelli M. (2008). Simple and complex deceits and ironies. *J. Pragmat.* 40 583–607. 10.1016/j.pragma.2007.05.004

[B13] Bosco F. M. Gabbatore I. (2017). Theory of mind in recognizing and recovering communicative failures. *Appl. Psycholinguist.* 38 57–88.

[B14] Bosco F. M. Gabbatore I. Tirassa M. (2014). A broad assessment of theory of mind in adolescence: the complexity of mindreading. *Conscious. Cogn.* 24 84–97. 10.1016/j.concog.2014.01.003 24491434

[B15] Bosco F. M. Gabbatore I. Tirassa M. Testa S. (2016). Psychometric properties of the Theory of Mind Assessment Scale in a sample of adolescents and adults. *Front. Psychol.* 7:566 10.3389/fpsyg.2016.00566 PMC4860419 27242563

[B16] Bosco F. M. Vallana M. Bucciarelli M. (2009). Comprehension of communicative intentions: the case of figurative language. *J. Cogn. Sci.* 10 245–272. 10.7334/psicothema2012.357

[B17] Bosco F. M. Vallana M. Bucciarelli M. (2012c). The inferential chain makes the difference between familiar and novel figurative expressions. *J. Cogn. Psychol.* 24 525–540. 10.1080/20445911.2012.658156

[B18] Brizio A. Gabbatore I. Tirassa M. Bosco F. M. (2015). “No more a child, not yet an adult”: studying social cognition in adolescence. *Front. Psychol.* 6:1011 10.3389/fpsyg.2015.01011 PMC4543799 26347664

[B19] Broomfield K. A. Robinson E. J. Robinson W. P. (2002). Children’s understanding about white lies. *Br. J. Dev. Psychol.* 20 47–65. 10.1348/026151002166316

[B20] Bucciarelli M. Colle L. Bara B. G. (2003). How children comprehend speech acts and communicative gestures. *J. Pragmat.* 35 207–241. 10.1016/S0378-2166(02)00099-1

[B21] Bussey K. (1999). Children’s categorization and evaluation of different types of lies and truths. *Child Dev.* 70 1338–1347. 10.1111/1467-8624.00098

[B22] Chandler M. Fritz A. S. Hala S. (1989). Small-scale deceit: Deception as a marker of two-, three-, and four-year-olds’ early theories of mind. *Child Dev.* 60 1263–1277. 10.2307/1130919 2612240

[B23] Cheung H. Siu T. S. C. Chen L. (2015). The roles of liar intention, lie content, and theory of mind in children’s evaluation of lies. *J. Exp. Child Psychol.* 132 1–13. 10.1016/j.jecp.2014.12.002 25576966

[B24] Colle L. Angeleri R. Vallana M. Sacco K. Bara B. G. Bosco F. M. (2013). Understanding the communicative impairments in schizophrenia: a preliminary study. *J. Commun. Disord.* 46 294–308. 10.1016/j.jcomdis.2013.01.003 23562700

[B25] Corcoran R. (2003). Inductive reasoning and the understanding of intention in schizophrenia. *Cogn. Neuropsychiatry* 8 223–235. 10.1080/13546800244000319 16571562

[B26] Corcoran R. Mercer G. Frith C. D. (1995). Schizophrenia, symptomatology and social inference. *Schizophr. Res.* 17 5–13. 10.1016/0920-9964(95)00024-G 8541250

[B27] Cummings L. (2015). Theory of mind in utterance interpretation: the case from clinical pragmatics. *Front. Psychol.* 6: 1286. 10.3389/fpsyg.2015.01286 PMC4549655 26379602

[B28] Demorest A. Meyer C. Phelps E. Gardner H. Winner E. (1984). Words speak louder than actions: understanding deliberately false remarks. *Child Dev.* 55 1527–1534. 10.2307/1130022

[B29] Dews S. Winner E. (1997). Attributing meaning to deliberately false utterances: the case of irony. *Adv. Psychol.* 122 377–414. 10.1016/S0166-4115(97)80142-2

[B30] Dews S. Winner E. Kaplan J. Rosenblatt E. Hunt M. Lim K. (1996). Children’s understanding of the meaning and functions of verbal irony. *Child Dev.* 67 3071–3085. 10.2307/1131767 9071771

[B31] Fernandez C. (2011). Mindful storytellers: emerging pragmatics and theory of mind development. *First Lang.* 33 20–46. 10.1177/0142723711422633

[B32] Filippova E. Astington J. W. (2008). Further development in social reasoning revealed in discourse irony understanding. *Child Dev.* 79 126–138. 10.1111/j.1467-8624.2007.01115.x 18269513

[B33] Filippova E. Astington J. W. (2010). Children’s understanding of social-cognitive and social- communicative aspects of discourse irony. *Child Dev.* 81 913–928. 10.1111/j.1467-8624.2010.01442.x 20573113

[B34] Flanagan O. J. (1992). *Consciousness Reconsidered.* Cambridge, MA: Mit Press.

[B35] Frith C. (1994). “Theory of mind in schizophrenia,” in *The Neuropsychology of Schizophrenia*, eds David A. S. Cutting J. C. (Hove: Erlbaum), 147–161.

[B36] Gabbatore I. Angeleri R. Bosco F. M. Cossa F. M. Bara B. G. Sacco K. (2014). Assessment of comunicative abilities in aphasic patients. *Minerva Psichiatr.* 55 45–55.

[B37] Garvey C. (1984). *Children’s Talk*, Vol. 21 Cambridge, MA: Harvard University Press

[B38] Grice H. P. (1989). *Studies in the Ways of Words.* Cambridge, MA: Harvard University Press.

[B39] Happé F. G. (1993). Communicative competence and theory of mind in autism: a test of relevance theory. *Cognition* 48 101–119. 10.1016/0010-0277(93)90026-R 8243028

[B40] Happé F. G. (1994). An advanced test of theory of mind: understanding of story characters’ thoughts and feelings by able autistic, mentally handicapped, and normal children and adults. *J. Autism Dev. Disord.* 24 129–154. 10.1007/BF02172093 8040158

[B41] Harris M. Pexman P. M. (2003). Children’s perceptions of the social functions of verbal irony. *Discourse Process.* 36 147–165. 10.1207/S15326950DP3603_1

[B42] Landis J. R. Koch G. G. (1977). The measurement of observer agreement for categorical data. *Biometrics* 33 159–174. 10.2307/2529310 843571

[B43] Langdon R. Coltheart M. (1999). Mentalising, schizotypy, and schizophrenia. *Cognition* 71 43–71. 10.1016/S0010-0277(99)00018-9 10394709

[B44] Lee K. (2000). “Lying as doing deceptive things with words: a speech act theoretical perspective,” in *Minds in the Making: Essays in Honor of David R. Olson*, ed. Astingtzon J. W. (Oxford: Blackwell), 177–196.

[B45] Leekman S. R. (1992). “Believing and deceiving: steps to becoming a good liar,” in *Social and Cognitive Factors in Early Deception*, eds Ceci S. J. Leichtman M. Putnick M. (Hillsdale, NJ: Erlbaum), 47–62.

[B46] Leinonen E. Letts C. Smith B. R. (2000). *Children’s Pragmatic Communication Difficulties.* London: Whurr Publishers.

[B47] Levinson S. C. (1983). *Pragmatics.* Cambridge: Cambridge University.

[B48] Lewis M. (1993). *The Lexical Approach*, Vol. 1 Hove: Language Teaching Publications, 993.

[B49] Loukusa S. Leinonen E. (2008). Development of comprehension of ironic utterances in 3-to 9-year-old finnish-speaking children. *Psychol. Lang. Commun.* 12 55–69. 10.2478/v10057-008-0003-0

[B50] Loukusa S. Moilanen J. (2009). Pragmatic inference abilities in individuals with Asperger syndrome or high-functioning autism. A review. *Res. Autism Spect. Dis.* 3 890–904. 10.1016/j.rasd.2009.05.002

[B51] Ma F. Evans A. D. Liu Y. Luo X. Xu F. (2015). To lie or not to lie? The influence of parenting and theory-of-mind understanding on three-year-old children’s honesty. *J. Moral Educ.* 44 198–212.

[B52] Mey J. (2001). *Pragmatics: An Introduction.* Oxford: Blackwell.

[B53] Nilsen E. S. Glenwright M. Huyder V. (2011). Children and adults understand that verbal irony interpretation depends on listener knowledge. *J. Cogn. Dev.* 12 374–409. 10.1080/15248372.2010.544693

[B54] Parola A. Gabbatore I. Bosco F. M. Bara B. G. Cossa F. M. Gindri P. (2016). Assessment of pragmatic impairment in right hemisphere damage. *J. Neurolinguistics* 39 10–25. 10.1016/j.jneuroling.2015.12.003

[B55] Perner J. (1991). *Understanding the Representational Mind.* Cambridge, MA: MIT Press.

[B56] Perner J. Leekam S. R. Wimmer H. (1987). Three-year-olds’ difficulty with false belief: the case for a conceptual deficit. *Br. J. Dev. Psychol.* 5 125–137. 10.1111/j.2044-835X.1987.tb01048.x

[B57] Perner J. Wimmer E. (1985). “John thinks, that Mary thinks that.” Attribution of second-order belief by 5- to 10-year-old children. *J. Child Exp. Psychol.* 39 437–471. 10.1016/0022-0965(85)90051-7

[B58] Peskin J. (1996). Guise and guile: children’s understanding of narratives in which the purpose of pretense is deception. *Child Dev.* 67 1735–1751. 10.2307/1131728

[B59] Pexman P. M. Glenwright M. (2007). How do typically developing children grasp the meaning of verbal irony? *J. Neurolinguistics* 20 178–196. 10.1016/j.jneuroling.2006.06.001

[B60] Polak A. Harris P. L. (1999). Deception by young children following noncompliance. *Dev. Psychol.* 35 561–568. 10.1037/0012-1649.35.2.561 10082026

[B61] Porter M. A. Coltheart M. Langdon R. (2008). Theory of mind in Williams syndrome assessed using a nonverbal task. *J. Autism Dev. Disord.* 38 806–814. 10.1007/s10803-007-0447-4 17874179

[B62] Premack D. Woodruff G. (1978). Does the chimpanzee have a theory of mind? *Behav. Brain Sci.* 1 515–526. 10.1017/S0140525X00076512

[B63] Reeder K. (1980). The emergence of illocutionary skills. *J. Child Lang.* 7 13–28. 10.1017/S0305000900007005 7372729

[B64] Russell J. Jarrold C. Potel D. (1995). What makes strategic deception difficult for children: the deception or the strategy. *Br. J. Dev. Psychol.* 12 301–314. 10.1111/j.2044-835X.1994.tb00636.x

[B65] Sacco K. Angeleri R. Bosco F. M. Colle L. Mate D. Bara B. G. (2008). Assessment battery for communication ABaCo: a new instrument for the evaluation of pragmatic abilities. *J. Cogn. Sci.* 9 111–115. 10.17791/jcs.2008.9.2.111

[B66] Searle J. R. (1975). “Indirect speech acts,” in *Syntax and Semantics: Speech Acts* Vol. 3 eds Cole P. Morgan J. L. (New York, NY: Academic Press), 59–82.

[B67] Sodian B. Frith U. (1992). Deception and sabotage in autistic, retarded and normal children. *J. Child Psychol. psychiatry* 33 591–605. 10.1111/j.1469-7610.1992.tb00893.x 1577901

[B68] Sperber D. Wilson D. (2002). Pragmatics, modularity and mind-reading. *Mind Lang.* 17 3–23. 10.1111/1468-0017.00186

[B69] Sullivan K. Winner E. Hopfield N. (1995). How children tell a lie from a joke: the role of second order mental state attributions. *Br. J. Dev. Psychol.* 13 191–204. 10.1111/j.2044-835X.1995.tb00673.x

[B70] Talwar V. Crossman A. (2011). From little white lies to filthy liars: the evolution of honesty and deception in young children. *Adv. Child Dev. Behav.* 40 139–179. 10.1016/B978-0-12-386491-8.00004-9 21887961

[B71] Talwar V. Gordon H. M. Lee K. (2007). Lying in the elementary school years: verbal deception and its relation to second-order belief understanding. *Dev. Psychol.* 43 804–810. 10.1037/0012-1649.43.3.804 17484589 PMC2597097

[B72] Talwar V. Lee K. (2008). Social and cognitive correlates of children’s lying behavior. *Child Dev.* 79 866–881. 10.1111/j.1467-8624.2008.01164.x 18717895 PMC3483871

[B73] Tirassa M. Bosco F. M. (2008). On the nature and role of intersubjectivity in human communication. *Emerg. Commun. Stud. New Technol. Pract. Commun.* 10 81–95. 10.1177/0003065114530156

[B74] Tirassa M. Bosco F. M. Colle L. (2006a). Sharedness and privateness in human early social life. *Cogn. Syst. Res.* 7 128–139. 10.1016/j.cogsys.2006.01.002

[B75] Tirassa M. Bosco F. M. Colle L. (2006b). Rethinking the ontogeny of mindreading. *Conscious. Cogn.* 15 197–217. 10.1016/j.concog.2005.06.005 16099174

[B76] Wellman H. M. Liu D. (2004). Scaling of theory-of-mind tasks. *Child Dev.* 75 523–541. 10.1111/j.1467-8624.2004.00691.x 15056204

[B77] Williams S. Moore K. Crossman A. M. Talwar V. (2016). The role of executive functions and theory of mind in children’s prosocial lie-telling. *J. Exp. Child Psychol.* 141 256–266. 10.1016/j.jecp.2015.08.001 26361741

[B78] Wimmer H. Perner J. (1983). Beliefs about beliefs: representation and constraining function of wrong beliefs in young children’s understanding of deception. *Cognition* 13 103–128. 10.1016/0010-0277(83)90004-5 6681741

[B79] Winner E. (1997). *The Point of Words: Children’s Understanding of Metaphor and Irony.* Cambridge MA, Harvard University.

[B80] Winner E. Leekman S. (1991). Distinguishing irony from deception: understanding the speaker’s second-order intention. *Br. J. Dev. Psychol.* 9 257–270. 10.1111/j.2044-835X.1991.tb00875.x

