# Supplementary material for: Sincere, Deceitful, and Ironic Communicative Acts and the Role of the Theory of Mind in Childhood
Source: Front Psychol. 2017 Jan 30;8:21. doi: 10.3389/fpsyg.2017.00021 (PMC5276808; doi:10.3389/fpsyg.2017.00021)
Supplement: Supplementary file 1 [file Data_Sheet_1.pdf]

Supplementary Material

Sincere, deceitful, and ironic communicative acts and the role of the Theory of Mind in childhood

Francesca M. Bosco [a,b] and Ilaria Gabbatore [a,c]

*** Correspondence:** Corresponding Author: ilaria.gabbatore@oulu.fi; ilariagabbatore@gmail.com.

1 Appendix A - Example of ABaCo items in the linguistic and extralinguistic scale.

	Comprehension tasks[a]	Production tasks[a]
**Linguistic Scale**		
**Sincere**	Frank, Paula and Claire are sitting at the table having dinner. Frank says: "Mmm, this pasta is delicious! Who made it?"<br>Paula replies: "I made it!"<br><br>- *What did the girl mean?*<br>If the participant repeats the actor's reply:<br>- *Who kooked the pasta?*	Mark and Caroline are on the couch reading magazines. At some point, Mark goes to the window and looks at the beautiful sunny day outside. Mark asks Caroline: What do you want to do this afternoon?<br><br>- *What could the girl say?*<br>(When in doubt): *What does that mean?*
**Deceit**	Ryan is enjoying some cookies, taking them from a small plate on the table. Hearing that his sister Julia is coming, Ryan pushes away the empty plate. Julia enters the room, looks at the empty plate, and asks: "Who finished my cookies?". Ryan replies: "I'm on a diet!"<br><br>- *What did the boy mean?*<br>If the participant repeats the actor's reply:<br>*What does it mean?*<br>- *Did the boy say the truth?*<br>- *Why did the boy say that to the girl?*	Richard is shaving in the bathroom when he drops a bottle of perfume in the sink. He quickly wipes his face and goes to his room. Shortly after, his sister Stephanie enters the room, shows him the empty bottle of perfume, and asks: "Who spilled my perfume?"<br><br>- *The boy does not want to be discovered. What could he say?*<br>(When in doubt): *What does that mean?*
**Irony**	Sarah and James are in a dress shop. Sarah is trying on a dress which is clearly too tight for her. Sarah asks James: "How does this fit me?" James replies: "Well, it looks kind of big for you!"<br><br>- *What did the boy mean?*<br>If the participant repeats the actor's reply:<br>*What does it mean?*<br>- *Was the boy serious when he said that?*<br>- *Why did the boy say that to the girl?*	Arianna - who is wearing her glasses on her head - is looking for something in the room, holding a book in her hand. She tries to read a page but can't do it without glasses. She asks Nathan: "Have you seen my glasses around?"<br><br>- *What could the boy say to be funny?*<br>(When in doubt): *What does that mean?*
**Extralinguistic Scale**		
**Sincere**	David is in the kitchen and has just finished cooking some pasta. He moves towards the door and looks into the study, where we can see Scarlett, sitting at the desk, intent on listening to some music through headphones and writing a letter. David shows her the plate and nods, also accompanied by a gesture with his arm, as if to say "Are you coming?" and Scarlett nods in assent.	Derek's car is parked in a deserted country lane. Derek looks as if he has been there a long time (he keeps looking at his watch, raising his hand above his eyes as if on the lookout for someone coming, he is on edge…). At last he sees a car coming…<br><br>- *The boy needs help. What gesture could he make?*<br>(When in doubt): *What does that mean?*

	- *In your opinion, what did the girl want to say to the boy?*<br>If the participant repeats the actor's reply:<br>- *Will the girl join the boy to eat?*	
**Deceit**	Naomi and Josh are arguing, having a pillow fight, in their bedroom. In all the confusion, Naomi hits the lamp on the bedside table, and it falls onto the floor. Having heard the noise, their dad comes to their room, puts his hands on his hips and with a questioning air and at the same time assuming a cross expression, as if to say "What's going on?", he points with his finger to the lamp on the floor. Naomi immediately picks up a book and shows it to her dad, as if to say "I was reading".<br><br>- *What did the girl want to say to her dad?*<br>If the participant repeats the actor's reply:<br>*What does it mean?*<br>- *Did the girl tell the truth?*<br>- *Why did the girl answer her dad with that gesture?*	Two little girls are playing in a yard. After a while Claire hits with her hand a vase of flowers that was standing on a table. Having heard the noise, her dad arrives and he puts his hands on his hips as if to ask her what has happened.<br><br>- *The girl doesn't want to be discovered. What gesture could she make?*<br>(When in doubt): *What does that mean?*
**Irony**	Peter and Alice are in the kitchen, sitting at a table that has been laid. Alice gets up to fetch a pan, which she brings to the table, and pours a ladle of soup into the dishes. They taste a spoonful and both pull a disgusted face, as if the soup were uneatable. Alice looks questioningly at Peter and Peter takes his fingers to his mouth and kisses his fingertips with an expression as if to say "Delicious!"<br><br>- *What did the boy want to say to the girl?*<br>If the participant repeats the actor's reply:<br>*What does it mean?*<br>- *Was he speaking seriously?*<br>- *Why did the boy answer the girl with that gesture?*	Alisha and John are in the kitchen emptying their shopping bags and putting everything in the cupboards. John absent-mindedly drops an egg he was about to put away. The egg breaks, making a mess on the table…<br><br>- *Imagine the girl wants to make fun of the boy. What gesture could she make?*<br>(When in doubt): *What does that mean?*

[a] <u>Sincere communication act:</u> In the *comprehension* tasks, the child obtains 1 point if he has recognized what the actor expressed and if he has understood what the utterance/gesture implies or presupposes. In the *production* tasks, the child obtains 1 point if he has produced a communication act (an utterance in the *linguistic* scale, a gesture in the *extralinguistic* scale), which is congruent with respect to the question.

<u>Deceit and irony:</u> In the *comprehension* tasks, the child obtains 1 point if he has understood not only what the actor said, but also that the actor communicated something ‘‘untrue” (through an utterance or a gesture, according to the scale) in order to lie (in the case of deceit) or in order to joke (in the case of irony).

In the *production* tasks, the child obtains 1 point if he produces a plausible act, fulfilling the requested goals. In the case of deceit, the subject has to communicate (through an utterance or a gesture, according to the scale) something that is not true with the purpose of deceiving. In the case of irony, the subject has to communicate (through an utterance or a gesture, according to the scale) something fun with the aim of joking.

2 Appendix B - Detailed description and examples from the first- and second-order tasks.

Task	Test question(s)	Scoring procedure
**Sally&Ann**<br>Two dolls, Sally and Anne, are introduced to the child: Sally has a basket in front of her, and Anne has a box. The child is presented with the following scenario: "Sally puts a ball in the basket. Then she goes out for a walk. While she is away, Anne takes the ball out of Sally's basket and puts it in her own box. After a while Sally returns.	"Test question": *Where does Sally think the ball is*?<br>"Justification question": *Why does Sally think the ball is there?*	A score of 1 was obtained when both the test and the justification answers were correct.
**Modified Smarties task**<br>The experimenter shows the box of a famous brand of chips to the children and asks 'What's in there?'. Then, experimenter opens the box and shows that the tube contains pencils rather than the expected chips.	Test question: *What someone else,who has not seen inside the tube, will think is in there before it is opened?*	A score of 1 was obtained when the children replied "chips".
**John &Mary:**<br>John and Mary are playing in the park when they notice the ice cream truck. Mary wants to buy an ice cream but she has no money. The ice cream man says Mary that she can go home to get some money since he was going to stay in the park all day long. So Mary goes home to get some money to buy an ice cream. Then, John sees the ice cream truck driving away and asks him where he is going. The ice cream man replies that he is going to the church to sell ice cream. Then John walks to his house to have lunch. Mary walks outside her home and meets the ice cream man, who tells her he is moving to the church. Later, John goes to Mary's house to play but Mary's mother tells him Mary went to get an ice cream. So John goes to find Mary.	"Second-order ignorance question": *Does John know that Mary knows where the ice cream truck is?*<br><br>"Second-order belief question": *Where does John go to look for Mary?*	Children's answers could be scored 1 (correct) or 0 (incorrect) for both the second- order ignorance and belief questions. The mean between the score obtained at the two test questions was run to perform the analyses.
**Maxi story**<br>Maxi and Bobby are in their kitchen when their mother brings in somechocolate. Maxi would like to have some chocolate and the mom tells him he can have some after he walks the dog. Unbeknown to Maxi but not to Bobby, the mother takes the chocolate to the neighbor's house. Unbeknown to Bobby, Maxi discovers that the mom has taken the chocolate to the neighbor's. Bobby then goes to look for Maxi outside. The mother tells him that Maxi has gone to get some chocolate.	"Second-order ignorance question": *'Does Bobby know that Maxi knows where the chocolate is?'*<br><br>"Second-order belief question": *'Where does Bobby go to look forMaxi?'*	Children's answers could be scored 1 (correct) or 0 (incorrect) for both the second- order ignorance and belief questions. The mean between the score obtained at the two test questions was run to perform the analyses.
**Picture Sequencing task**<br>Stories were depicted in 4-card picture sequences, with a simple black and white cartoon style. Two types of stories were used in the present study:<br><br>- 4 False-belief stories:<br>A person, unaware of an event in a story, acts on a false belief.<br>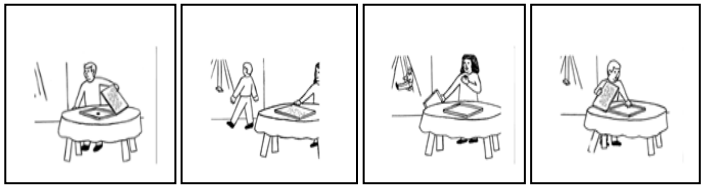<br><br>- 2 Social-script stories:<br>More than one person interacting in everyday social routines.<br>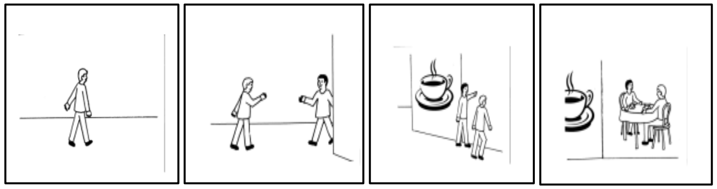	Instruction given to the child:<br>"When we are ready to start, I will ask you to turn the cards over; your task is to line the cards up in a straight line, like a comic-strip. You need to arrange the cards in the correct order so that they show a logical sequence of events."	Score range was 0-6: A sequence scored 2 points if the first card was positioned correctly, 2 points if the last card was positioned correctly and 1point each for the second and third cards being positioned correctly. Failure to produce a sequence scored 0.
